# The Role of Hydrogen in Decarbonizing U.S. Iron and Steel Production

**DOI:** 10.1021/acs.est.4c05756

**Published:** 2025-03-06

**Authors:** Katherine H Jordan, Paulina Jaramillo, Valerie J Karplus, Peter J Adams, Nicholas Z Muller

**Affiliations:** † Engineering and Public Policy, Carnegie Mellon University, 5000 Forbes Ave, Pittsburgh, Pennsylvania 15213, United States; ‡ Wilson E. Scott Institute for Energy Innovation, Carnegie Mellon University, 5000 Forbes Ave, Pittsburgh, Pennsylvania 15213, United States; § Civil and Environmental Engineering, Carnegie Mellon University, 5000 Forbes Ave, Pittsburgh, Pennsylvania 15213, United States; ∥ Tepper School of Business, Carnegie Mellon University, 5000 Forbes Ave, Pittsburgh, Pennsylvania 15213, United States; ⊥ National Bureau of Economic Research, 1050 Massachusetts Avenue, Cambridge, Massachusetts 02138, United States

**Keywords:** industrial decarbonization, energy systems modeling, steel, blast furnace, direct reduced iron, hydrogen, carbon capture

## Abstract

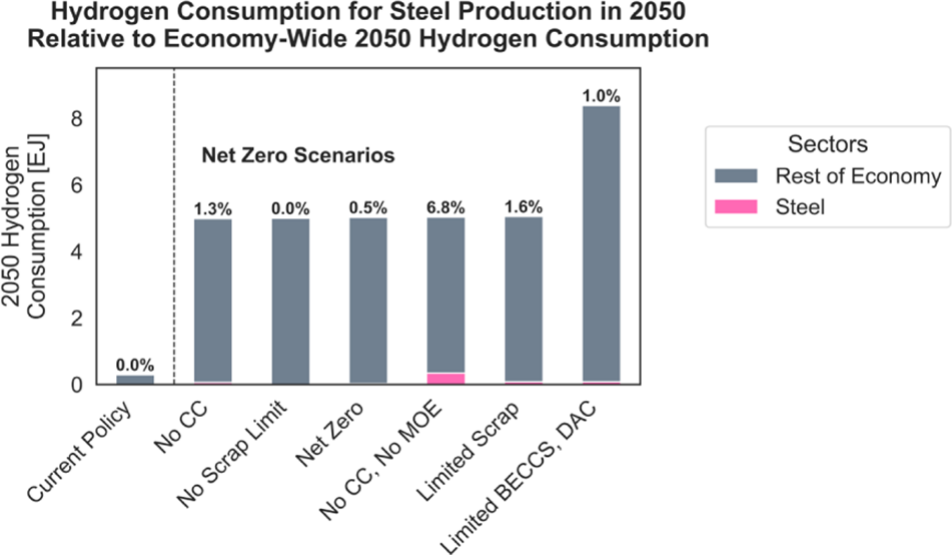

This study investigates the role of hydrogen as a decarbonization strategy for the iron and steel industry in the United States (U.S.) in the presence of an economy-wide net zero CO_2_ emissions target. Our analysis shows that hydrogen-based direct reduced iron (H_2_DRI) provides a cost-effective decarbonization strategy only under a relatively narrow set of conditions. Using today’s best estimates of the capital and variable costs of alternative decarbonized iron and steelmaking technologies in a U.S. economy-wide simulation framework, we find that carbon capture technologies can achieve comparable decarbonization levels by 2050 and greater cumulative emissions reductions from iron and steel production at a lower cost. Simulations suggest hydrogen contributes to economy-wide decarbonization, but H_2_DRI is not the preferred use case for hydrogen in most scenarios. The average abatement cost for U.S. iron and steel production could be as low as $70/tonne CO_2_ with existing technologies plus carbon capture, while the cost with H_2_DRI rises to over $500/tonne CO_2_. We also find that IRA tax credits are insufficient to spur hydrogen use in steelmaking in our model and that a green steel production tax credit would need to be as high as $300/tonne steel to lead to sustained H_2_DRI use.

## Abstract 2

This work shows that carbon capture may be a more economical choice than hydrogen to decarbonize steel production when we explicitly consider other competing demands for hydrogen.

## 1 Introduction

The industrial sector accounts for nearly a quarter of U.S. greenhouse gas emissions, ranking third after transportation and electricity generation.^1^ The Energy Information Administration forecasts that by 2024, industrial emissions could overtake electricity generation emissions as the electricity sector deploys additional renewable generation sources. At the same time, industry will also likely electrify some processes.^2^ Specific heavy industries, such as iron and steel production, present significant challenges and are often labeled “difficult to decarbonize”.^3^ Iron and steel account for approximately 95% of U.S. metal production by mass and 10% of U.S. industrial carbon dioxide (CO_2_) emissions.^1,3^ Further, the U.S. is the world’s fourth-largest steel-producing country, behind China, India, and Japan, although only 4% of the world’s steel is produced in the U.S.^2,4^ While the U.S. accounts for a small share of global steel production, we must reduce emissions from domestic production to meet net-zero targets. Anticipated growth in domestic steel demand, driven by materials needed for renewable energy infrastructure, further underscores the sector’s importance.^5^

In this study, we simulate decarbonizing U.S. iron and steel production in the context of economy-wide net-zero targets. Many studies explore decarbonizing iron and steel production in isolation, but little attention has been paid to decarbonizing this sector within the broader energy economy. In particular, an existing literature focuses on enabling hydrogen direct reduced iron production, but these studies largely do not consider the potential competing uses of electricity or hydrogen. This broader logic is underscored by a recent publication in Joule by Shafiee and Schrag (2024),^6^ which examines potential applications of hydrogen in a net-zero transition. This study finds that uses may be much more constrained than conventional wisdom suggests. As such, it is critical to explore decarbonization of steelmaking, and other hard-to-abate sectors, in the context of economy-wide decarbonization.

Globally, most steel is produced in integrated steel mills, which rely on three primary emissions-intensive process steps: coke-making, ironmaking, and steelmaking. Coal is heated at high temperatures in an oxygen-free environment to create coke, then added to a blast furnace (BF) with iron ore and limestone to produce molten iron. The molten iron is moved to a basic oxygen furnace (BOF) and combined with scrap steel. Then, oxygen is added to remove carbon and impurities, producing molten steel. All process steps combined produce ∼2,200 kg CO_2_ per tonne steel, primarily driven by BF emissions. BFs are particularly challenging to decarbonize because carbon is used to chemically reduce the iron ore. While integrated steel production is the primary steel-producing route globally, it accounts for only about 30% of US-made steel.

In the US, roughly 70% of steel is produced in electric arc furnaces (EAFs), and some projections suggest that as early as 2050, 50% of steel may be EAF-made globally.^7^ An EAF can be fed with scrap steel, direct reduced iron, or both. Direct reduced iron (DRI) is produced by adding pelleted iron ore to a rotary kiln or shaft furnace. Then, a reducing agent is added to reduce the iron ore. In the US, most DRI units use natural gas as both a heating source and a reducing agent, but it is possible to use hydrogen. The DRI is then fed to the EAF, typically blended together scrap steel depending on the purity requirements of the targeted steel product. The scrap steel adds a carbon source, but if additional carbon is needed, carbon-containing materials may be added to the EAF.^8^ The emissions intensity strongly depends on the feedstock (scrap steel vs DRI) and the carbon intensity of the electricity used, ranging from 420–1,950 kg CO_2_ per tonne steel.^9^ Using 100% scrap requires less energy and emits less CO_2_ relative to making and using high quantities of DRI.

A widely studied decarbonization option for BF-BOF steel is carbon capture (CC). While CC has not yet been deployed for blast furnaces, it has been successful in other industries.^10^ Tata Steel commissioned a CC unit on the BF at its Jamshedpur Works steel production facility and ArcelorMittal is designing a CC system with the ability to capture 50–70% of the CO_2_ emitted in BF gas.^11^ The International Energy Agency (IEA) characterizes the importance of capture applied to blast furnace gas as “very high” for meeting global net-zero emissions.^12^

EAF steel fed with scrap and powered by zero-emission electricity can be near zero-emission. High-quality scrap steel theoretically reduces the need for DRI, but scrap supply is finite, and impurities compound with recycling over time. Several pathways exist to decarbonize the DRI process. CO_2_ can be captured from the top gas of a natural gas-based DRI furnace. This process is more straightforward than CC on a BF, as the off-gas is richer in CO_2_.^9^ To date, two industrial-scale DRI steelmaking facilities use CC, Emirates Steel’s Al Reyadah Emirates plant and Ternium’s Guerrero plant.^9,13^ Further, Nucor is retrofitting a DRI with CC in Louisiana.^14^ Hydrogen-based DRI (H_2_DRI) has also received substantial attention from steel producers, policymakers, and researchers. Using green hydrogen and an EAF powered by zero-emission electricity brings steelmaking emissions to near zero.^15−21^ Several steelmakers have already begun exploring this pathway, including ArcelorMittal, Hybrit, H2 Green Steel, Tenva, ThyssenKrupp, and Saltzgitter, although none has demonstrated sustained 100% hydrogen use.^15^ H_2_DRI-EAF steel has minor chemical process emissions, equaling approximately 160 kg CO_2_/tonne steel, plus CO_2_ emissions from residual natural gas used for process heat.^22^

Novel iron and steel production methods are emerging. In molten oxide electrolysis (MOE), an electric current is applied to liquid iron oxide containing an inert anode, producing liquid iron and pure oxygen.^23^ While not yet proven at commercial scales, startup Boston Metal aims to have a demonstration plant operating by 2024 and commercialization by 2026.^24^ MOE steel powered by zero-emission electricity would have near-zero emissions, but the Boston Metal design does include limited natural gas consumption for process needs. Some chemical process emissions would remain (∼80 kg CO_2_/tonne steel).^25^ Several studies have also explored biomass as a replacement for coke in blast furnaces, but the CO_2_ abatement potential is highly sensitive to life-cycle emissions factors, such as land use change and cultivation practices.^9^

Our analysis simulates a range of decarbonization options with varying technology-readiness levels (TRLs). The International Energy Agency (IEA) estimates that blending H2 into natural-gas based DRI has a TRL of 7, while 100% H2DRI has a TRL of 5 and DRI with CC has a TRL of 9. Electrolytic technologies, including molten oxide electrolysis and electrowinning, have an estimated TRL of 4. We do not explicitly simulate electrowinning as a technology pathway in this analysis. While distinct from MOE, both technologies are expected to have high upfront capital costs and consume high levels of electricity. As such, similar modeling conditions will lead to the deployment of either technology, so we opt only to model MOE. More detailed assessments comparing electrolytic steelmaking technologies exist, but are outside the scope of this analysis.^26^

A wide range of academic literature and reports from government agencies and industrial experts discuss potential future pathways for iron and steel decarbonization. The DOE has produced several reports dedicated to industrial decarbonization. The Industrial Decarbonization Roadmap and Pathways to Commercial Liftoff studies both emphasize that achieving near-zero GHGs from steel production will likely require a diverse range of solutions, including energy efficiency improvements, H2DRI, CCS (on both DRI and BF technologies), and industrial electrification, predicated on further industrial scale demonstrations and technological breakthroughs.^27,28^

While several studies conduct detailed techno-economic analysis (TEA) to individual iron and steel decarbonization technologies, few analyses robustly model iron and steel decarbonization pathways within the broader energy system.^28,29^ Examples of detailed TEA studies include Elsheikh and Eveloy, who investigate renewables-based H2DRI in five geographical locations with varying onsite and grid sourced electricity characteristics. Their localized assessment incorporates high spatial and temporal resolution, finding that a 10% cost increment could yield 70% emissions reduction relative to conventional DRI steelmaking. Rosner et al.’s TEA explores what hydrogen procurement price would be necessary for H2DRI to directly compete with natural gas, finding that H2 price would need to fall to $1.63–$1.70/kg.^55^ Key decarbonization pathways involve electricity, hydrogen, and carbon capture, with feasibility hinging on the sector’s competitiveness against other end uses. This is especially pertinent for hydrogen, given its broad potential as a decarbonization strategy and the limited current green hydrogen production. The optimal hydrogen applications for effective emissions reduction across the economy remain uncertain.

Kim et al. undertake a systematic review of iron and steel decarbonization, including identifying remaining research gaps. Specifically, the authors highlight a need for researchers to explicitly consider interconnections between iron and steel and other sociotechnical systems.^30^ While our analysis does not simulate macroeconomic interdependencies, we do account for competing demands for electricity, hydrogen, and domestic storage of captured carbon.

Energy systems optimization models (ESOMS) are a valuable tool to simulate future decarbonization pathways. These models simulate deployment of technologies defined by their capital and operating costs, emissions factors, energy consumption, and in-use lifetime. ESOMs optimize economy-wide system costs while accounting for interactions across the energy economy. However, most existing models do not capture granularity in iron and steel production technologies, nor do they simulate deep decarbonization.

Prior studies focus either on the iron and steel industry only or on specific technologies. Mayer et al. use a computable general equilibrium (CGE) model of the European Union to simulate iron and steel production with green hydrogen, capturing macroeconomic impacts associated with the transition and simulating multiple energy sectors.^31^ Their analysis focuses on the transition from the BF-BOF steel pathway to either hydrogen DRI-EAF or electrolytic direct steel production. However, their work does not explore economy-wide decarbonization. Moglianesi et al. use a global bottom-up optimization model to simulate the effects of technological learning on iron and steel technology deployment in The Netherlands. But, Moglianesi et al. do not model decarbonization policies and thus simulate limited iron and steel emissions reductions.^32^ Sánchez Diéguez et al. developed an open-source bottom-up energy system optimization model, IESA-Opt, with technological representation in multiple industrial sectors.^33^ The authors characterize several iron and steel decarbonization pathways, including electrolytic production, direct reduction with hydrogen, and blast furnace CCUS, but does not represent electric arc furnaces. Furthermore, Sánchez Diéguez et al.’s analysis focuses on energy system-wide trends, and thus, the discussion of iron and steel is limited. The IEA’s Iron and Steel Technology Roadmap simulates iron and steel deployment within an energy systems model, but the global scope and focus on countries with more steel production (India and China) make extrapolation of insights to the U.S. context challenging.^12^ Further, the IEA and other existing studies do not include the impact of the Inflation Reduction Act (IRA).^34^ Durga, Speizer, and Edmonds use the Global Change Analysis Model to simulate alternative iron and steel production technologies, finding that carbon capture plays a larger role in iron and steel decarbonization than hydrogen-based technologies in a net-zero scenario, but this analysis does not consider more novel technologies like molten oxide electrolysis.^35^

In this study, we simulate multiple decarbonization pathways for U.S. iron and steel production, including H_2_DRI, carbon capture, and molten oxide electrolysis. We do so using an economy-wide ESOM, Temoa, against the backdrop of U.S. economy-wide decarbonization policies. We seek to understand (1) how provisions of the IRA could affect the deployment of iron and steel decarbonization technologies, (2) what iron and steel decarbonization technologies contribute to a least-cost net-zero emission energy system, and (3) how the availability of particular technologies changes the contribution of the iron and steel industry to economy-wide decarbonization.

## 2 Materials and Methods

### 2.1 Model and Database

This study simulates the U.S. energy system using a bottom-up, open-source energy system optimization model (ESOM), the Tools for Energy Model Optimization and Analysis (Temoa).^36^ Bottom-up ESOMs like Temoa require detailed techno-economic parameters for technologies across the energy economy. Temoa’s objective function minimizes system-wide present value of the costs of operating the energy system. Modeled costs include fuel, capital, fixed, and variable costs for energy conversion technologies. Similar modeling tools include MARKAL/TIMES models, OSeMOSYS, and MESSAGE.^37−39^ However, unlike many other ESOMs, Temoa is entirely open-source.

The database used in this analysis represents the U.S. energy system as six distinct sectors: electricity generation, fuel supply, transportation, commercial buildings, residential buildings, and heavy industry. We represent the continental U.S. as nine regions, and the model’s time horizon runs from 2020 to 2050 in five-year time steps. The model solves myopically, optimizing one period at a time without foresight into future year’s constraints, technology availability, or changes to technology costs. Solving the model myopically allows for a more detailed representation of the energy system. For example, when the model is run myopically, we are able to use a database with hourly temporal resolution for eight representative days in a given year. Hourly resolution allows for a more accurate representation of technological capabilities, particularly in the power sector. Modeling myopically does limit the model’s ability to “see” future constraints, but in some ways, this is more realistic than a model that optimizes based on constraints out to 2050 (or beyond). Decision makers do not know how the policy landscape will evolve and oftentimes must make investments with imperfect information. In both myopic and perfect foresight modeling, existing capacity carries over from one time period to the next. The model will only invest in new capital if (a) the existing capacity has reached the end of its lifetime and retires (b) it is less expensive to replace old, inefficient equipment with new, more efficient equipment or (c) the model is forced to retire capacity to meet an exogenous constraint, like an emissions limit.

The database includes detailed techno-economic characterization of technologies across the energy system. We model hydrogen production via steam methane reforming with and without carbon capture, electrolysis, and bioenergy with carbon capture. We use an exogenous projection of fossil fuel prices from the Annual Energy Outlook (AEO) for most fuels, but Temoa calculates electricity prices endogenously and biomass costs are drawn from a supply curve.^40^ We also simulate two carbon dioxide removal (CDR) technologies: electricity production from bioenergy with carbon capture and storage (BECCS) and direct air capture (DAC). The base dollar year is 2018 real USD and we assume a 5% real discount rate. More comprehensive documentation of the model’s structure and the technologies encompassed within each sector can be accessed in our repository and the database used to generate results can be found in a Zenodo repository.^41,42^

### 2.2 Iron and Steel Representation

We draw data on energy consumption for coke ovens, blast furnaces, basic oxygen furnaces, electric arc furnaces, and direct reduced iron production from the Department of Energy’s Bandwidth Study on Energy Use and Potential Energy Saving Opportunities in U.S. Iron and Steel Manufacturing.^43^ We characterize the age, production capacity, and physical location of existing iron and steel facilities based on the Global Energy Monitor’s steel plant tracker.^44^ We allow existing plants to be retrofitted with carbon capture technology. We draw carbon capture parameters from Panja et al. and assume a 90% capture rate for DRI and a 65% capture rate on blast furnaces.^45^ Processes requiring nonenergy feedstocks include feedstock costs in their variable operating costs. In addition to the steel production technologies, we represent CO_2_ emissions and energy consumption from coke production, steel casting, and hot and cold rolling. As Temoa is not a full life-cycle model, the system boundaries do not include mining, transportation, or further downstream processing. We assume that all crude steel is hot-rolled and that 30% of hot-rolled steel is cold-rolled.^43,46^Tables S1 and S2 provide cost and energy consumption assumptions.

We draw steel demand from 2020 to 2050 from Princeton University’s Net-zero America report (see the Supporting Information for annual demand values).^47^ To our knowledge, regional or state-level open-source steel demand data do not exist. As such, while all other demands in Temoa are regionalized, we model a national demand for steel. In practice, this means that steel can be manufactured anywhere in the U.S. to meet the national demand. If regionally disaggregated data becomes available, this characterization will be updated. Based on historical average consumption, we limit scrap steel consumption to 55 MMT per year.^48^

### 2.3 Inflation Reduction Act Representation

The Inflation Reduction Act (IRA) drastically changed the U.S. energy policy landscape. The most relevant provisions are the clean hydrogen production tax credit (45 V) and the carbon capture credit (45Q). We also represent the production and investment tax credits (PTC, ITC) for new renewable generation, the PTC for existing nuclear generation, and tax credits for zero-emission passenger and commercial vehicles. While not directly relevant to the industrial sector, policies targeting one sector of the economy may impact iron and steel production. For example, reduced renewable electricity prices may make electrolytic hydrogen more favorable, spurring a more rapid transition to H_2_DRI. The SI provides details of IRA modeling.

### 2.4 Scenario Overview

This work simulates diverse scenarios to explore the costs and emissions from different iron and steel decarbonization technology pathways. First, we model a current policy case, which assumes no new major energy or climate legislation after the Inflation Reduction Act. We subsequently model several economy-wide net-zero CO_2_-equivalent emissions scenarios. Temoa represents CO_2_ emissions from the entire energy system and CH_4_ emissions (expressed as CO_2_-e) from upstream methane leakage from the natural gas system. We distinguish “zero” from “net-zero,” as net-zero allows for positive emissions as long as they are offset by emissions removal technologies: bioenergy with carbon capture and sequestration (BECCS) or direct air capture (DAC). In each net-zero case, we enforce a linear decrease from 2020 emissions levels to net-zero in 2050.

The first net-zero case allows all iron and steel technology options. Next, we test three cases where various decarbonization options are unavailable to explore what would happen if technologies become constrained or do not mature as expected. We examine a case with no carbon capture on blast furnaces. We also simulate a case with no carbon capture on BFs or DRI production and a case without iron and steel CC or MOE. We then simulate a scenario without limits on available steel scrap and one where steel scrap availability is constrained beyond historical availability. The latter simulates the case where scrap demand from other countries or industries restricts availability or copper content in recycled scrap becomes too high for reuse.^49^ Lastly, we force more direct decarbonization by banning BECCS. In this scenario, we also ban direct air capture until 2040 but allow limited DAC in 2045 and 2050, as system-wide net-zero is currently infeasible in Temoa without some carbon dioxide removal.

## 3 Results and Discussion

### 3.1 Iron and Steel Production

**Figure 1:**
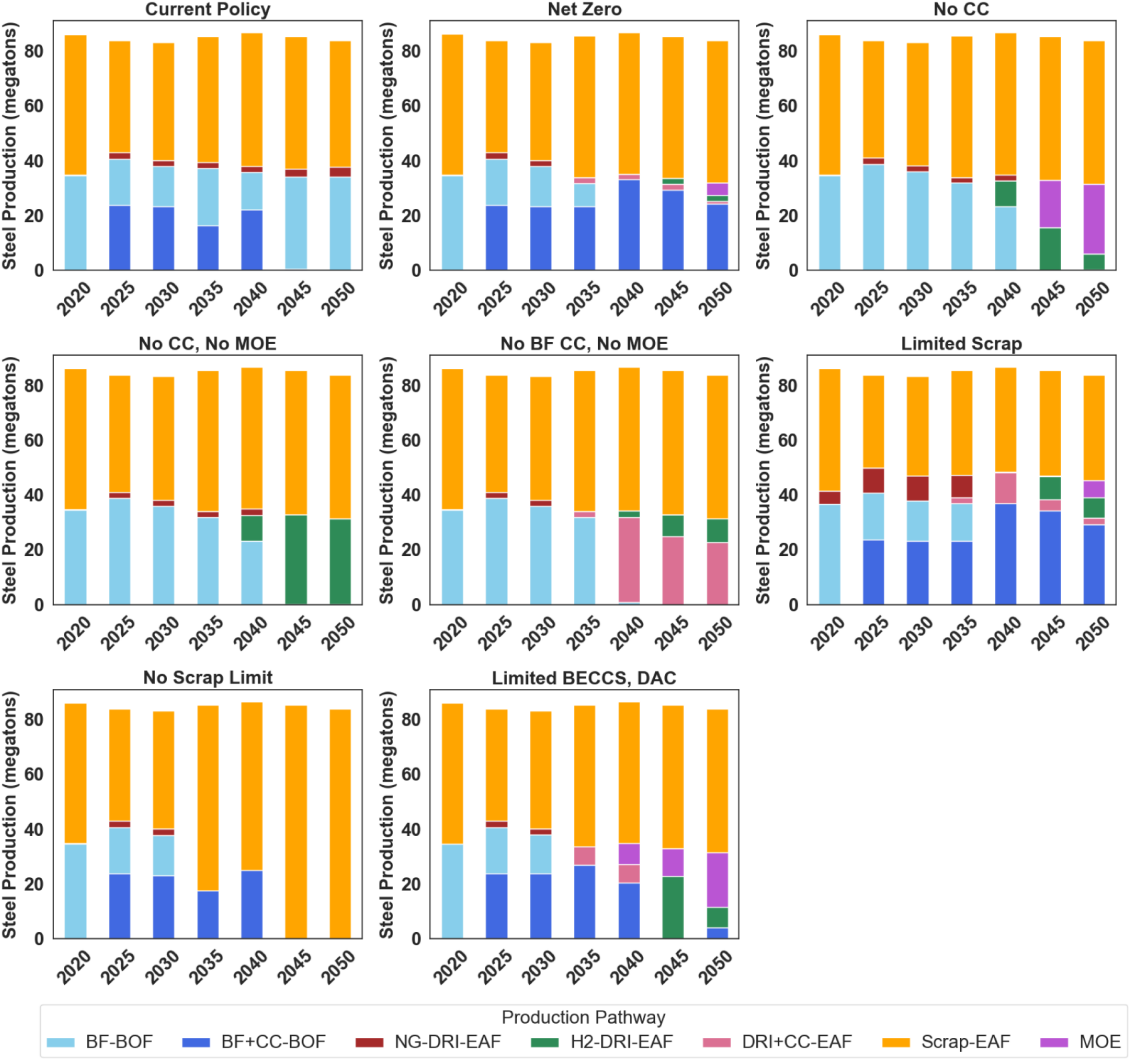
Steel production by source. From left to right, row 1: Current policy, least-cost net-zero, net-zero no carbon capture. Row 2: Net-zero no carbon capture no MOE, net-zero no blast furnace carbon capture no MOE, net-zero limited scrap. Row 3: Net-zero no limits on scrap production, net-zero no BECCS + limited DAC.

Figure 1 presents steel production under the modeled scenarios. In the current policy, the model deploys carbon capture on some blast furnace capacity in early periods, driven by the IRA’s 45Q tax credits. In this scenario, natural gas-based DRI-EAF without CC expands to about 4 megatons of steel annually, just under 5% of total production.

In most of the net-zero scenarios, scrap-EAF and BF+CC-BOF methods dominate steel production throughout the analysis period, with small amounts of steel produced using other technologies. Our findings do not indicate a clear “winner takes all” among these technologies, as regional outcomes vary due to differences in commodity prices such as natural gas and electricity. Additionally, the model optimizes economy-wide deployment of hydrogen and CC to maximize the benefits of the 45 V and 45Q tax incentives and to meet emissions targets. The model also includes constraints that limit the speed of hydrogen infrastructure deployment. Consequently, hydrogen is allocated where it offers the greatest cost advantage. This could lead to the transition of some sectors to 100% hydrogen use, while the remaining hydrogen is distributed at lower levels across the economy.

Our results indicate that, under current cost assumptions and with all technologies available, DRI is not the most cost-effective option under an economy-wide net-zero constraint. Despite residual emissions from blast furnaces with CC (modeled with a 65% carbon capture rate), the model finds this pathway as the least cost. In the scenarios where carbon capture is unavailable for BFs or DRI, the model turns to MOE and H_2_DRI. The model does not adopt high levels of H_2_DRI except when it is the only remaining decarbonization option characterized (i.e., no CC, no MOE). It is worth noting again that the Temoa framework assumes a central decision-maker that optimizes the decisions for the entire energy system. In reality, many actors will make decarbonization decisions and participate in multiple markets. As a result, a steelmaking facility may face financial conditions that encourage the deployment of H_2_DRI. For example, a facility could negotiate a hydrogen purchasing agreement at a low cost or face regulatory constraints in building a CO_2_ pipeline. The results of our modeling do not preclude situations where buyers may find it attractive to invest in H_2_DRI, but that when decarbonization is viewed through a central planner framework, H_2_DRI deployment is limited.

**Figure 2:**
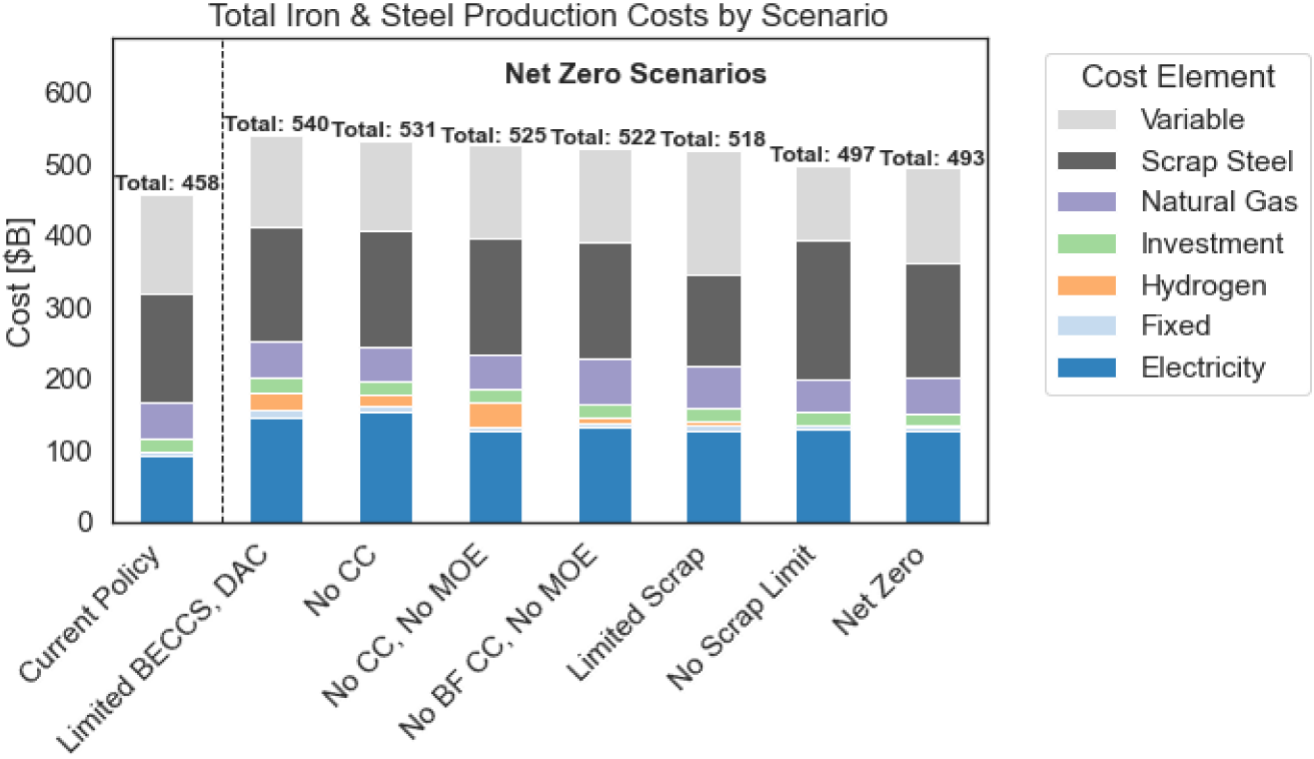
Cumulative discounted costs of steel production by scenario. Costs by scenario and production method can be found in Figure S1. “Variable” refers to other variable costs, such as materials beyond scrap steel.

Figure 2 displays steel production costs by component, quantifying the economic driver(s) behind decarbonization technology adoption. The figure demonstrates that electricity, natural gas, scrap, and other variable costs (primarily material costs) dwarf the fixed and other investment costs for new facilities.

Over the model’s time horizon, steel production in the least-cost net-zero scenario costs about 7% more than the current policy. This is equal to approximately 0.15% of 2020 U.S. Gross Domestic Product (GDP). Steel production in the most expensive net-zero scenario is roughly 20% more expensive than in the current policy.

In scenarios banning carbon capture, costs increase due to increased electricity, hydrogen, and scrap steel consumption. While carbon capture systems do increase electricity consumption relative to unabated DRI or BF use, it is less than the increase required for MOE (MOE requires 22× the electricity per tonne crude steel that a CC retrofit consumes). In the scenarios that deploy H_2_DRI, the increased costs from hydrogen consumption are greater than the increased electricity costs for operating the carbon capture unit. Both electricity and hydrogen costs are calculated endogenously within Temoa and depend on the production source.

**Figure 3:**
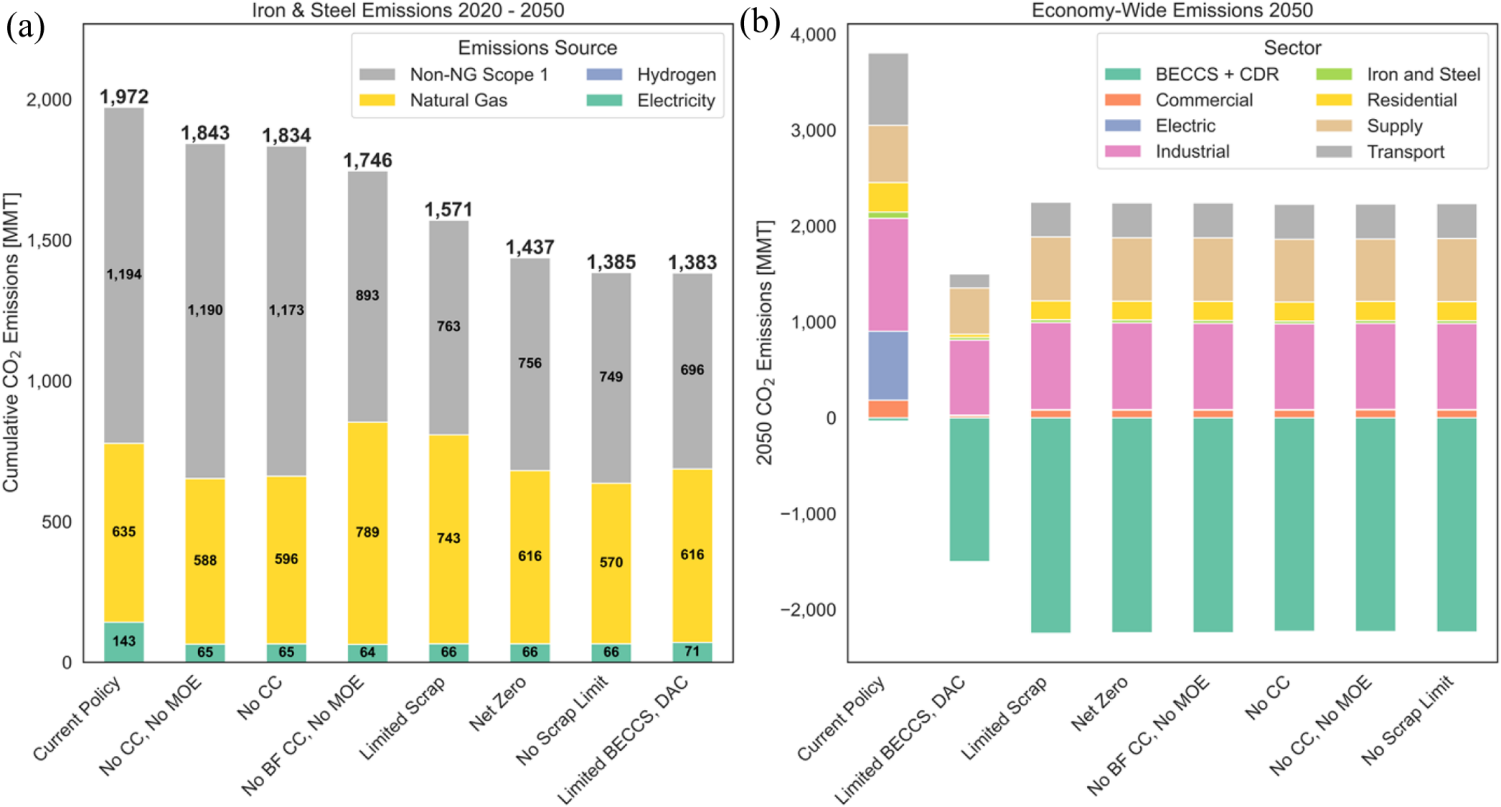
(a) Cumulative CO_2_ emissions by source from iron and steel production. “Non-NG Scope 1” emissions are non-natural gas process emissions, including emissions from coke and emissions from oxygen combusting carbon to CO, which later oxides to CO_2_. (b) 2050 economy-wide CO_2_ emissions by sector. “Supply” refers to upstream fuel supply emissions.

Figure 3a shows cumulative greenhouse gas emissions from iron and steel production across the model’s time horizon. In each case, the scope 1 emissions from natural gas combustion comprise the largest share of emissions. Further, more than 50% of emissions are non-natural gas scope 1, including emissions from coke combustion, limestone/fluxes, and oxygen oxidizing carbon to CO_2_. Despite driving costs, electricity is not a major contributor to cumulative emissions due to decreasing grid emissions driven by the IRA and the falling cost of renewable electricity generation. In net-zero scenarios that use hydrogen for steel production, hydrogen does not contribute to emissions, as it is produced using zero-emission electrolysis or BECCS-to-hydrogen. Iron and steel production emissions decrease in all net-zero scenarios but do not reach zero. Figure 3b displays economy-wide emissions in 2050 to contextualize iron and steel emissions in the broader economy and to demonstrate the direct emissions reduction that occurs in the Limited BECCS, DAC scenario.

**Figure 4:**
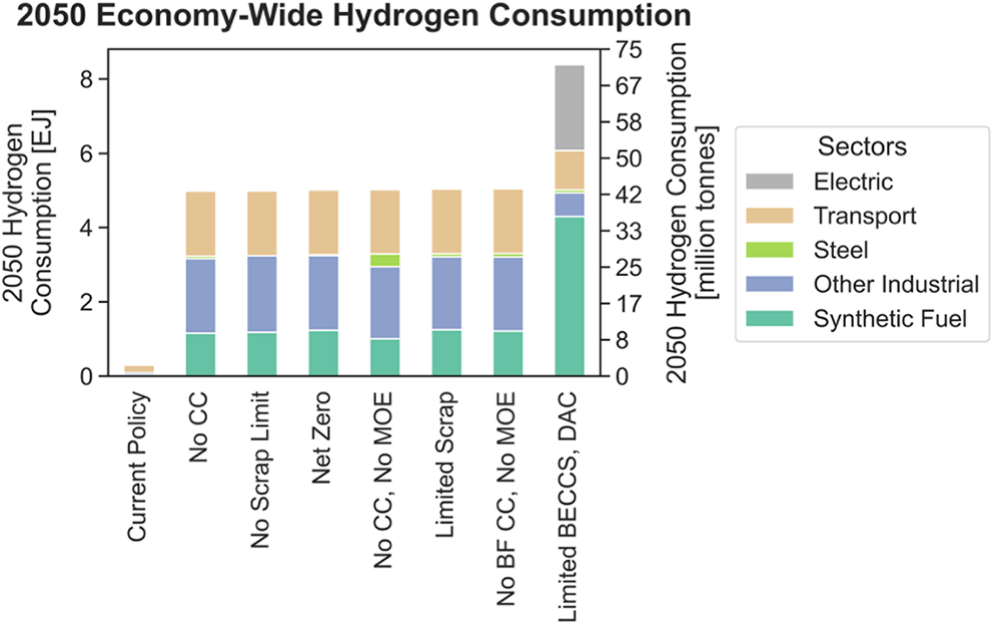
Economy-wide hydrogen consumption by end-use. “Transport” includes only hydrogen used directly in fuel cells, while “Synthetic Fuels” are hydrogen reacted with carbon dioxide to produce drop-in replacements for fossil sources and may be used in any sector of the economy. “Other Industrial” includes all industrial hydrogen consumption beyond what is used by the iron and steel industry.

Despite limited H_2_DRI deployment, the model uses hydrogen as a decarbonization strategy elsewhere in the economy. Cumulative economy-wide hydrogen consumption ranges from 80 to 110 exajoules (670–920 MMT) in the various net-zero cases. Figure 4 shows hydrogen consumption in 2050. Hydrogen is predominantly used for heavy-duty fuel cell vehicles, industrial process heat, and synthetic fuels for aviation, shipping, and commercial and residential buildings. In the scenario with limited BECCS and DAC deployment, the model generates more hydrogen than in the other net-zero cases, with most of the additional H_2_ used for industrial process heat or combusted to generate electricity.

Table 1 reports cumulative avoided emissions, the percent change in annual emissions between 2020 to 2050, and the average CO_2_ abatement cost for steel production. Of the net-zero scenarios explored, the least cost net-zero (with no scrap limits), and net-zero with no BECCS and limited DAC avoid the most CO_2_ relative to the current policy (535, 587, and 589 million metric tons (MMT), respectively). Emissions from iron and steel in 2050 are ∼60% lower than 2020 in all net-zero scenarios, including the limited DAC and BECCS scenario. However, the wide range in cumulative avoided emissions has implications for limiting the increase in global temperatures to 1.5 °C above preindustrial levels without overshoot.^50^

**Table 1:** Cumulative Avoided Emissions from Iron and Steel Production^a^

scenario	percent change in iron and steel emissions, 2020–2050	cumulative avoided iron and steel emissions 2020–2050 [MMT]	average abatement cost, iron and steel [$/tonne CO_2_]
current policy	–5%		
limited scrap	–59%	401	150
cost optimal net-zero	–59%	535	70
net-zero, no CC	–61%	138	530
net-zero, no BF+CC, no MOE	–58%	226	280
net-zero, no CC, no MOE	–60%	129	520
net-zero, no scrap limit	–60%	587	70
net-zero, limited BECCS, DAC	–61%	589	140

a As the scenarios have different technologies available, there is not a systematic relationship between the average abatement cost and the cumulative avoided emissions.

In the least cost net-zero scenario, the model deploys high levels of carbon capture on blast furnaces starting in 2025 due to the IRA and continues to expand CC capacity through the end of the time horizon. In technologically constrained scenarios, the model waits until the CO_2_ emissions constraint is more stringent to deploy decarbonization strategies, missing out on near-term decarbonization. This may be partially attributable to Temoa’s myopic formulation; it solves one period at a time and does not “know” about the increasingly strict emissions limit. A myopic approach approximates behavior where steelmakers only respond to incentives once they are implemented.

The least-cost net-zero and net-zero with no scrap limit scenarios have the lowest average abatement cost of all scenarios, at ∼ $70/tonne CO_2_. Average abatement costs are as high as $530/tonne CO_2_ in the scenarios in which technology options are limited.

### 3.2 Promoting Green Hydrogen-Based DRI

In light of the growing interest in H2DRI as a key decarbonization strategy for iron production, we conducted simulations involving a green steel production tax credit specifically designed for 100% green hydrogen-based DRI. These simulations encompassed tax credits ranging from $100 to $400 per tonne of steel, aiming to determine when Temoa prefers deploying H2DRI as a decarbonization strategy. The credits are available throughout the model’s time horizon.

**Figure 5:**
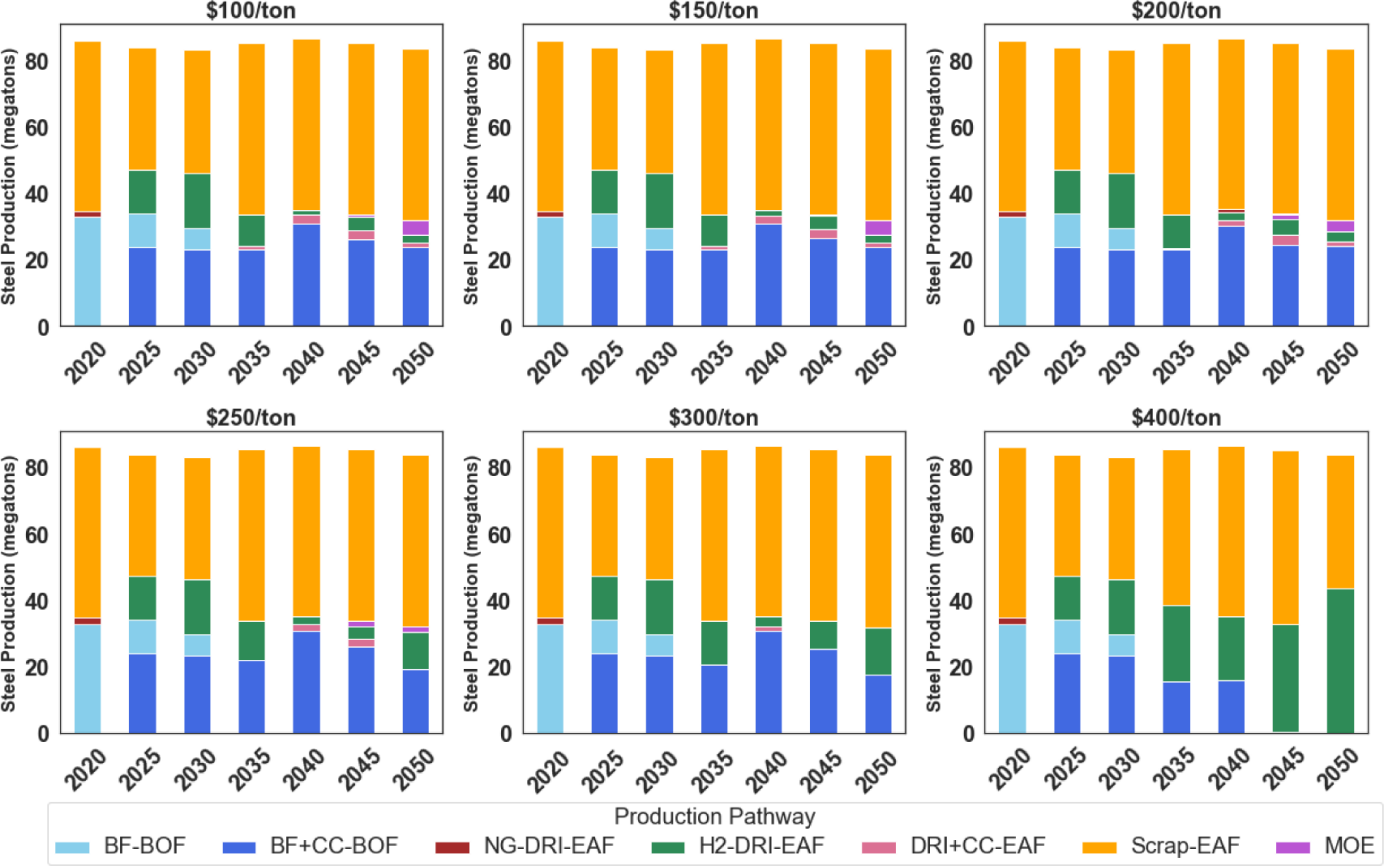
Steel production by pathway under rising green steel PTCs for green hydrogen-based DRI. All scenarios include a linear emissions constraint from 2020 emissions levels to net-zero in 2050.

Figure 5 illustrates the resulting production pathways. Green steel PTCs ranging from $100/tonne to $200/tonne, combined with the IRA’s green hydrogen PTC, led to H2DRI deployment between 2025 and 2039. However, at these PTC levels, H2DRI+EAF production decreased to less than 5% of annual steel production after the IRA credits expired. This scenario is likely unrealistic, as steel producers would typically not abandon DRI assets before their useful lifetime ends. Instead, they might opt for steam methane-reformed hydrogen or natural gas, especially in flexible DRI furnace designs, if there are no policy incentives to encourage ongoing zero-emission hydrogen use.

Because capital costs represent such a small portion of the overall expense (as shown in Figure 2), the model finds that abandoning hydrogen-based DRI altogether is the cost-optimal pathway. While this may not align with realistic industry behavior, these modeling results offer valuable insights. They indicate that the IRA combined with a $100 to $200/tonne PTC effectively stimulates H_2_DRI adoption, but this pathway loses cost competitiveness after the IRA program ends.

The IRA may lead to hydrogen-based technology cost reductions due to economies of scale and learning (which we do not model), but it also may be the case that these technologies are no longer cost competitive without continued policy support or drastic cost reductions. The results emphasize the potential need for clean technology support beyond the IRA and for additional analyses on the long-term effects of the IRA. Once the PTC reaches $300/ton, the model more consistently uses H_2_DRI. However, it is not until a PTC of $400/tonne that H_2_DRI becomes the dominant pathway. $400/tonne would represent a considerable subsidy; the average steel price in Q2 2023 was $658/tonne.^51^^1^

Our results demonstrate that hydrogen-based DRI is not the cost-optimal decarbonization pathway for steelmaking in the U.S. when we consider decarbonization across the energy economy, despite current research and industry focus on this pathway. Rather, we find that carbon capture reaches a similar level of decarbonization by 2050 and achieves even greater cumulative emissions reductions compared to more novel technologies due to its earlier deployment. The climate responds to cumulative GHG emissions, so this distinction is critical as it has implications for the ability to meet temperature targets without overshoot. It is important to note that the U.S. has large CO_2_ storage resources that are well-characterized. Other steel-producing countries may not have as good storage potential, which limits the generalizability of our results to the global iron and steel sector.

Our results demonstrate that the average cost of iron and steel emissions abatement when H_2_DRI-EAF is the only available option is about 5 times higher than the case where all options are modeled. The case allowing DRI+CC-EAF, but not BOF-BF+CC, costs about 4 times more. The overall discounted cost for iron and steel production from 2020 to 2050 is ∼ $32 billion more in the no carbon capture, no MOE scenario than the least-cost net-zero scenario.

Our findings add to the ongoing discussion surrounding the optimal pathway(s) to decarbonize iron and steel production. The DOE’s Pathways to Commercial Liftoff report estimated that BF-BOF+CC would cost $500–600 per ton liquid steel, compared to $550–800 per ton for H_2_DRI-EAF. They concluded that some CCS would likely be needed alongside clean hydrogen to decarbonize the sector, but did not account for competing hydrogen demands.^28^ Further, a 2024 study found that H_2_DRI-EAF increased costs by 79% when using green hydrogen, compared to a 7% increase for DRI+CC-EAF (relative to conventional steel technology).^53^

Hydrogen is a potential decarbonization strategy for numerous industries. It could be used in fuel cell vehicles, to generate synthetic fuels, as a blending agent with natural gas, and combusted to produce electricity. Currently, hydrogen is expensive and energy-intensive to produce, leaving questions about where it may be best used to reach net-zero GHG emissions economy-wide. When a range of hydrogen uses across the economy is modeled, hydrogen-based DRI is not a preferred use case. Rather, hydrogen is preferentially used in the transportation sector and for industrial process heat and boilers.

To explore the potential efficacy of a green steel PTC to encourage economic H_2_DRI deployment, we model subsidies ranging from $100–$400 per tonne of steel produced using hydrogen from zero-emissions electricity. A $100/tonne credit incentivizes the model to deploy H_2_DRI while the IRA is active, but it ceases production when the 45 V tax credits sunset. While this result is likely not behaviorally realistic, it demonstrates the need for continued policy support for hydrogen-based technologies and further research on the long-term learning rate impacts of the IRA.

Under an economy-wide net-zero constraint, annual GHG emissions from the iron and steel industry fall ∼60% from 2020 to 2050, demonstrating trade-offs between achieving a net-zero energy economy and decarbonizing a single sector. While some sectors, like electricity generation, completely decarbonize, our results show that it may be less expensive for some steel emissions to be offset through CDR. However, CDR technologies remain unproven at a commercial scale. Furthermore, bringing GHG emissions to net-zero is not the only desirable target. For example, remaining emitting processes will have negative air quality impacts on the communities where they are located. Some manufacturers have also committed to sourcing net-zero steel to decarbonize their supply chains, indicating a demand for deeply decarbonized steel, which may be perceived at odds with relying on CDR to cover residual emissions.^54^

This study provides insights into the conditions under which hydrogen can contribute to U.S. iron and steel decarbonization. Under our current assumptions, a PTC of $300/tonne of steel or more would be necessary to spur large-scale H_2_DRI deployment. Our results may be impacted by the model’s myopic formulation; it is possible that the model would adopt decarbonization technologies such as H_2_DRI earlier or more aggressively if it optimized all time periods simultaneously. However, a myopic scenario is somewhat representative of reality. The U.S. does not currently have a policy enforcing net-zero GHG emissions and it is uncertain whether one will be adopted.

It is important to emphasize that the results presented in this paper are specific to the U.S. context. Other countries may lack the resources for large-scale geologic CO_2_ sequestration, may be able to develop low-carbon hydrogen infrastructure more rapidly, or may have fewer sectors competing for hydrogen. Under such conditions, hydrogen-based direct reduced iron (DRI) could emerge as the preferred option in a system-wide optimization model. Furthermore, while modeling iron and steel decarbonization in an economy-wide model allows for system-level insights, such as how hydrogen may compete across sectors, there are several limitations to our approach. First, simulating technology-level detail in an economy-wide model necessitates making simplifying assumptions to reduce computational demands. While we draw our technoeconomic parameters from a range of industry and literature sources, the economics of steelmaking technology will vary by subnational region, time of deployment, and facility scale, whereas our model simulations focus on the U.S. as a whole. There is also substantial uncertainty in the assumed cost trajectory of technologies such molten oxide electrolysis and electrolysis-based production of hydrogen. Similarly, the cost of carbon capture and sequestration (CCS) is also uncertain, especially given limited deployment at scale. We estimate total geologic sequestration potential using data from the USGS, but the exact magnitude of available storage volume remains uncertain, as does the storage longevity when the CO_2_ is stored as a gas, not mineralized. Future work should explore these uncertainties in more detail.

Temoa, as a bottom-up model, does not consider international trade. However, according to the Congressional Research Service, the share of direct steel consumption in the U.S. supplied by mills located in the U.S. ranged from 70% to 90% between 2010 and 2021^1^. Today, steel policy in the U.S. includes tariffs, and import substitution is not likely to be politically acceptable as part of a steel industry decarbonization strategy in the U.S. Temoa does not include end-use materials efficiency or other trends that could reduce end-use demand for steel., As a result, steel production in the United States remains relatively flat, as it has for the past several decades, excluding the years of the Global Financial Crisis. Changes to domestic manufacturing trends, economic growth or recession, evolving trade relations, or radical improvements in materials efficiency could all lead to changes in domestic production, but these are beyond the scope of this analysis.

While further research is needed, our findings challenge the notion that hydrogen-based direct reduction provides a cost-optimal solution for decarbonizing the iron and steel sector in the U.S. when considering alternative uses of hydrogen in an economy-wide perspective. These results are striking; they indicate that, despite the attention and financing dedicated to H_2_DRI, it is likely not the least-cost for attaining economy-wide net-zero emissions by 2050, unless substantial technology improvements and cost reductions occur, some of the other decarbonization options underperform or become unavailable, or transformational technologies become available to decarbonize other sectors, which would free up green hydrogen for use in DRI. Barring major shifts in cost, our results foresee an important role for CCS to decarbonize integrated steelmaking and potentially also for NG-DRI with CC, if BF-CC proves infeasible due to the difficulties posed by legacy plant layouts and configurations. Only if CC is not available and the scrap supply is limited do we see a major role for hydrogen in the production of DRI in the US, which in turn may limit the role of the iron and steel industry in the achievement of the U.S. net zero goal by midcentury.

## Supporting Information Available

Input parameters for iron and steel technology characterization, supplemental figures, and IRA modeling (PDF)

## Footnotes

1 Temoa’s base dollar year is 2018. The average steel price in Q2 2023 was 794 USD 2023, which was discounted to 2018 USD using a CPI of 1.206.^52^
