# Supplementary material for: The Role of Hydrogen in Decarbonizing U.S. Iron and Steel Production
Source: Environ Sci Technol. 2025 Mar 6;59(10):4915–25. doi: 10.1021/acs.est.4c05756 (PMC11924224; doi:10.1021/acs.est.4c05756)
Supplement: Supplementary file 1 — es4c05756_si_001.pdf [file es4c05756_si_001.pdf]

Supplemental Information

Title: The Role of Hydrogen in Decarbonizing US Iron and Steel Production

Authors: Katherine H Jordan[1*]; Paulina Jaramillo[1]; Valerie J Karplus[1,2]; Peter J Adams[1,3] & Nicholas Z Muller[1,4,5].

Affiliations: [1]Engineering and Public Policy, Carnegie Mellon University, 5000 Forbes Ave, Pittsburgh, PA, 15213, USA
[2] Wilson E. Scott Institute for Energy Innovation, Carnegie Mellon University, 5000 Forbes Ave, Pittsburgh, PA, 15213, USA
[3]Civil and Environmental Engineering, Carnegie Mellon University, 5000 Forbes Ave, Pittsburgh, PA, 15213, USA
[4]Tepper School of Business, Carnegie Mellon University, 5000 Forbes Ave, Pittsburgh, PA, 15213, USA
[5]National Bureau of Economic Research, 1050 Massachusetts Avenue, Cambridge, MA, 02138.
*Corresponding author contact: khjordan@andrew.cmu.edu

Pages: 8
Figures: 3
Tables: 4

I. Input parameters for iron and steel characterization

Tables S.1, S.2, and S.3 provide input assumptions for iron and steel characterization in Temoa. All monetary values are provided in USD 2018, as this is the base dollar year in Temoa.

Table S.1: Energy consumption in iron & steel production by source

**Process**	**Energy Carrier**	**Energy Consumption**	**Units [input per unit output]**	**Source**
Coke Oven	Natural Gas	0.39	PJ/Mt	U.S. DOE [1]
	Electricity	0.12		
	Coke oven gas	7.2		
Blast Furnace	Coke	0.471	Mt/Mt	U.S. DOE [1]
	Natural Gas	1.88	PJ/Mt	
	Coal	2.13		
Blast Furnace CC Retrofit	Electricity	0.56	PJ/Mt	Panja et al. [2]
Basic Oxygen Furnace	Natural Gas	0.43	PJ/Mt	U.S. DOE [1]
	Electricity	0.13		
Direct Reduced Iron Production	Natural Gas	10.4	PJ/Mt	U.S. DOE [1]
	Electricity	0.31		
DRI CC Retrofit	Electricity	0.65	PJ/Mt	Panja et al. [2]
Electric Arc Furnace	Natural Gas	1.65	PJ/Mt	U.S. DOE [1]
	Electricity	0.51		
Molten Oxide Electrolysis	Natural Gas	1.21	PJ/Mt	Netherlands Organisation for Applied Scientific Research [3]
	Electricity	13.33	PJ/Mt	
Steel Casting	Natural Gas	0.09	PJ/Mt	U.S. DOE [1]
	Electricity	0.08		
Hot Rolling	Natural Gas	2.52	PJ/Mt	U.S. DOE [1]
	Electricity	0.44		
Cold Rolling	Natural Gas	1.84	PJ/Mt	U.S. DOE [1]

	Electricity	1.22		

Table S.2: Cost parameters and year assumed available for iron and steel production technologies in Temoa.

Process	Capital Cost	Variable Cost	Fixed Cost	Unit	Source	Year available
	**$M/(unit production /year)**	**$M/(unit production/ year)**	**$M/unit capacity**			
Blast Furnace	305	209	–	Mt pig iron	IEA [4]	2020
BF CCS retrofit	30	18	3	Mt steel	Panja et al. [2]	2025
Basic Oxygen Furnace	145	57	7	Mt steel	IEA [4]	2020
DRI	223	198	–	Mt DRI	IEA [4]	2020
DRI CCS retrofit	38	24	3	Mt DRI	Panja et al. [2]	2020
Electric Arc Furnace	124	49	–	Mt steel	IEA [4]	2020
Molten oxide electrolysis	841	126	68	Mt steel	Netherlands Organisation for Applied Scientific Research [3]	2035
Steel Casting	No costs, as costs will be the same (only one technology option)					2020
Hot Rolling						2020
Cold Rolling						2020

Table S.3: Input commodity prices

Commodity	Price	Source
Lump Ore	$85 / ton	Germeshuizen & Blom [5]
Sintered Ore	$135 / ton	
Pelletized Ore	$135 / ton	

Limestone / Fluxes	$45 / ton	
Scrap Steel	$250 / ton	Statista, compiled from USGS [6]
Oxygen	$0.12 / Nm^3	Intratec [7]

All input commodities, including scrap steel, have a flat price. Temoa is not a general equilibrium model, and thus does not represent supply and demand relationships. While we model some commodities in other industries with a supply curve, we did not have sufficient data to do so for input commodities to steelmaking.

Table S.4: Steel demand, drawn from Princeton's Net Zero America Report [8]

**Year**	**Steel demand [MMt]**
2020	86.1
2025	83.8
2030	83.2
2035	85.4
2040	86.6
2045	85.3
2050	83.8

In addition to direct emissions from iron and steel production, our analysis includes upstream emissions from coal extraction and natural gas production. For upstream coal production, we assume 0.44 kt CO_2-equivalent per PJ of coal, drawn from table 1b of Argonne's GREET model (v1.8) [9]. The emissions factor includes emissions from energy use due to extraction, coal-bed methane, and coal transportation. Similarly, we assume a 2.3% methane leakage rate on a volume basis, consistent with Alvarez et al. [53]. To convert methane emissions to CO_2-equivalent units, we use a 100-year global warming potential of 34 g CO_2-e/g CH_4, consistent with the Environmental Protection Agency's estimate of 28 – 36 g CO_2-e/g CH_4 [54].

II. Supplemental figures

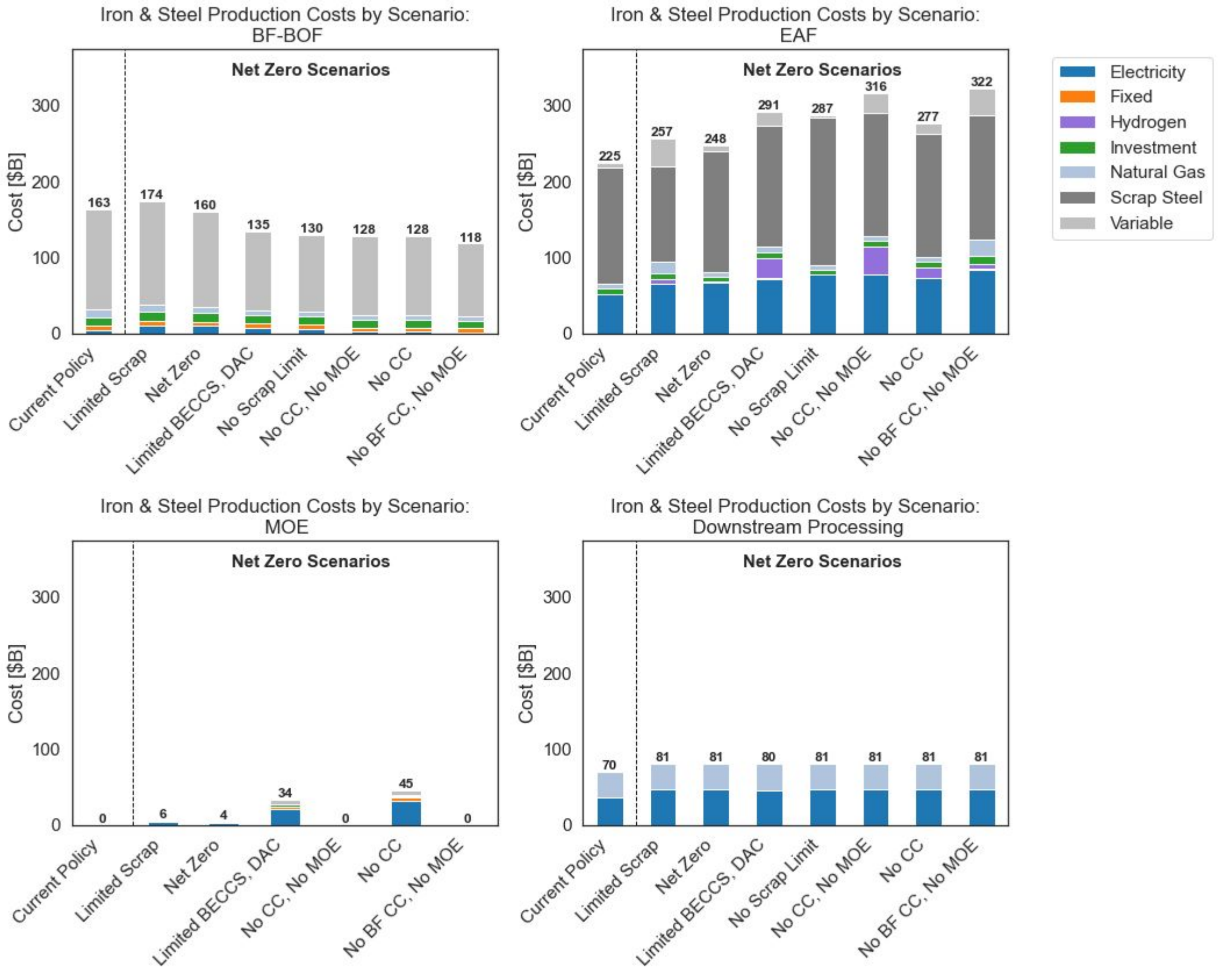

Figure S.1: Discounted cumulative iron & steel costs by production pathway. Downstream costs reported separately.

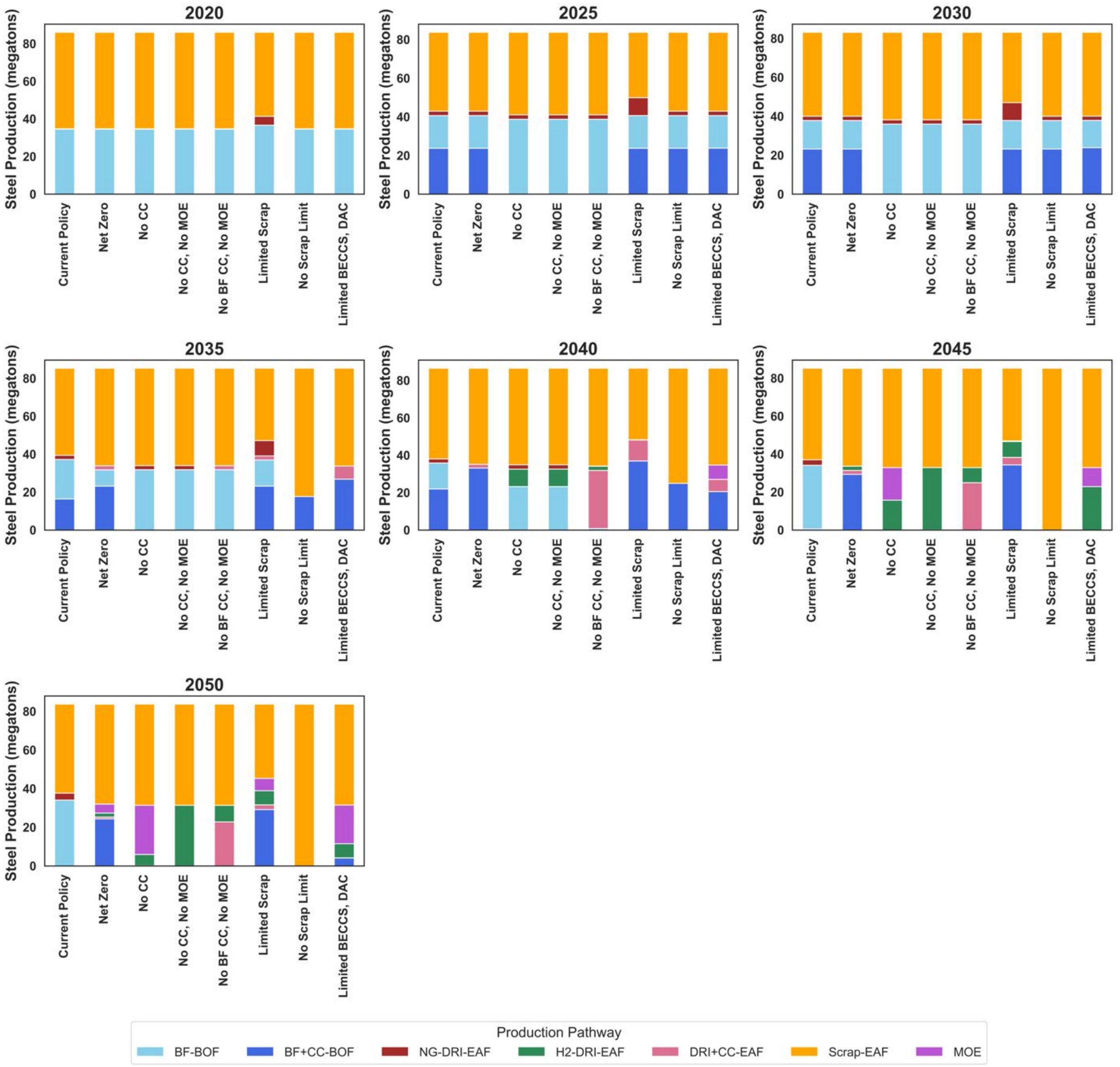

Figure S.2: Steel production by source (identical data to Figure 1, but with policy scenarios on the x-axis and the subplots as years)

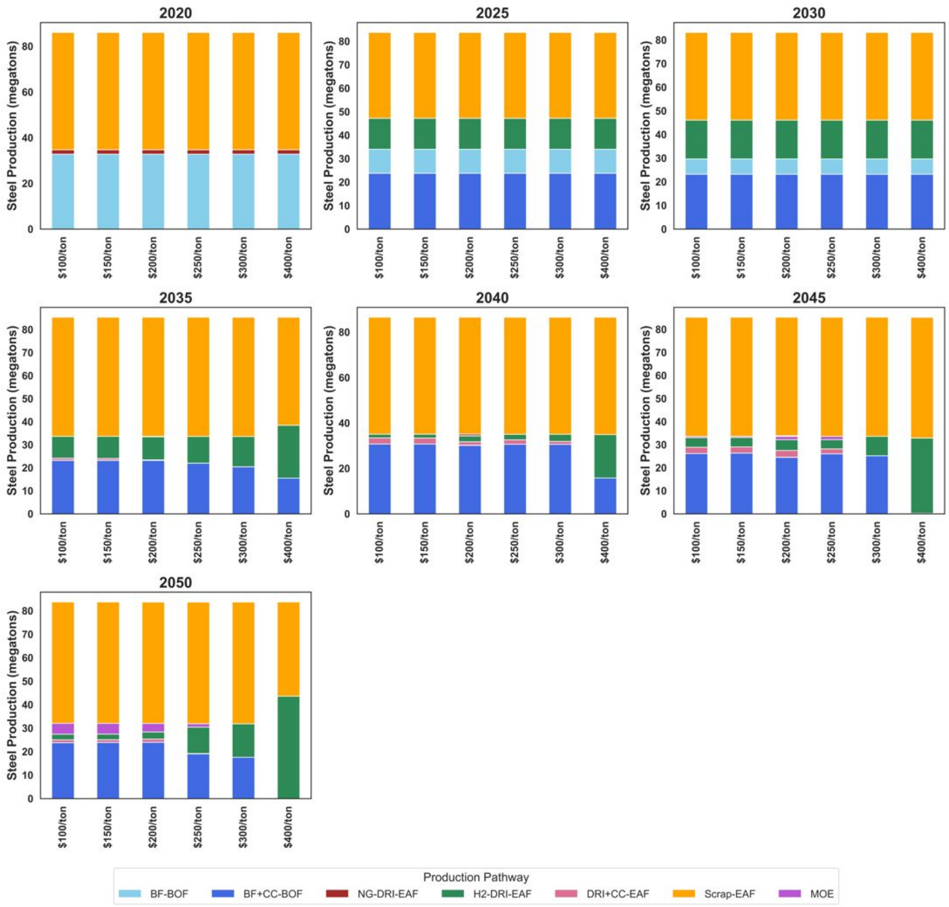

Figure S.3: Steel production by pathway under rising green steel PTCs for green hydrogen-based DRI. All scenarios include a linear emissions constraint from 2020 emissions levels to net-zero in 2050. (Identical data to Figure 5, but with policy scenarios on the x-axis and the subplots as years)

III. IRA modeling

All scenarios included in this analysis incorporate major provisions of the IRA. The two most relevant provisions are the credits for clean hydrogen production (45V) and carbon capture (45Q). We simulate a simplified version of the 45V tax credits. In reality, the tax credit is tiered, with increasing credits for decreased carbon intensity. Recent U.S. Treasury guidance suggests adopting a "three pillars" structure, where qualifying hydrogen resources must have hourly load

matching, loads must occur in the same region as the electricity resource (deliverability), and only be powered by new clean generation that would not otherwise have been installed (additionality) [10]. This analysis assumes hourly matching but does not enforce additionality or deliverability for electrolytic hydrogen. We do require all qualifying electrolytic hydrogen resources to consume electricity from new, zero-emission electricity, but these electric generators can meet other demands and may have been constructed without electrolytic H_2 demand. We allow hydrogen produced with new zero-emission electricity capacity or biomass with CC to qualify for a tax credit. We assume hydrogen produced by either of these pathways would qualify for the full $3/kg credit. We also model hydrogen produced by steam methane reforming (SMR) of natural gas. The International Energy Association assumes an emission factor of 1.0 kg CO_2/kg H_2 for natural gas reforming with carbon capture, qualifying for the second tier of the tax credit ($1/kg H2); thus, we allow SMR+CC hydrogen to receive a $1/kg credit [11]. 45Q is simpler to implement; we assume any captured CO_2 from natural gas DRI + CC or BF + CC receives $85/tonne sequestered CO_2. For a comprehensive overview of all modeled IRA provisions, see Jordan et al., Appendix A [12].

IV. References

[1] K. Jamison, C. Kramer, S. Brueske, and A. Fisher, "Bandwidth Study on Energy Use and Potential Energy Saving Opportunities in U.S. Iron and Steel Manufacturing," US Department of Energy, Washington, DC., Jun. 2015.

[2] P. Panja, B. McPherson, and M. Deo, "Techno-Economic Analysis of Amine-based CO2 Capture Technology: Hunter Plant Case Study," *Carbon Capture Science & Technology*, vol. 3, Jun. 2022, doi: https://doi.org/10.1016/j.ccst.2022.100041.

[3] K. West, "Technology factsheet: high-temperature molten oxide electrolysis steelmaking (UCOLYSIS)," Netherlands Organisation for Applied Scientific Research, Amsterdam, Sep. 2020. Accessed: Nov. 12, 2023. [Online]. Available: https://energy.nl/media/data/Ulcolysis-Technology-Factsheet_080920.pdf

[4] L. Van Wortswinkel and W. Nijs, “IEA ETSAP Technology Brief I02-Iron and Steel,” IEA Energy Technology Systems Analysis Programme, May 2010. [Online]. Available: https://iea-etsap.org/E-TechDS/PDF/I02-Iron&Steel-GS-AD-gct.pdf
[5] L. M. Germeshuizen and P. W. E. Blom, “A techno-economic evaluation of the use of hydrogen in a steel production process, utilizing nuclear process heat,” *International Journal of Hydrogen Energy*, vol. 38, no. 25, pp. 10671–10682, Aug. 2013, doi: 10.1016/j.ijhydene.2013.06.076.
[6] B. Aleves, “Iron and steel scrap: U.S. prices 2022,” Statista. Accessed: Jan. 03, 2024. [Online]. Available: https://www.statista.com/statistics/209362/iron-and-steel-prices-in-the-us/
[7] Intratec, “Oxygen Price.” Accessed: Jan. 03, 2024. [Online]. Available: https://www.intratec.us/products/water-utility-costs/commodity/oxygen-price
[8] A. Pascale and E. D. Larson, “Princeton’s Net-Zero America study Annex J: Iron and Steel Industry Transition,” Aug. 2021. [Online]. Available: https://netzeroamerica.princeton.edu/img/NZA%20Annex%20J%20-%20Iron%20&%20steel%20industry.pdf
[9] M. Wang, Y. Wu, and A. Algowainy, “Argonne GREET Publication : Operating Manual for GREET: Version 1.7,” Argonne National Laboratory, Feb. 2007. Accessed: Nov. 03, 2024. [Online]. Available: https://greet.anl.gov/publication-ycrv02rp
[10] “U.S. Department of the Treasury, IRS Release Guidance on Hydrogen Production Credit to Drive American Innovation and Strengthen Energy Security,” U.S. Department of the Treasury. Accessed: Jan. 03, 2024. [Online]. Available: https://home.treasury.gov/news/press-releases/jy2010
[11] IEA, “The Future of Hydrogen,” International Energy Agency, Jun. 2019. [Online]. Available: https://iea.blob.core.windows.net/assets/9e3a3493-b9a6-4b7d-b499-7ca48e357561/The_Future_of_Hydrogen.pdf
[12] K. Jordan, P. Adams, P. Jaramillo, and N. Muller, “Closing the Gap: Achieving U.S. climate goals beyond the Inflation Reduction Act,” *Renewable and Sustainable Energy Transitions*, vol. 4, Aug. 2023, doi: 10.1016/j.rset.2023.100065.
